# Plasma miRNA Profile of Crohn’s Disease and Rheumatoid Arthritis Patients

**DOI:** 10.3390/biology11040508

**Published:** 2022-03-25

**Authors:** Tatiana D. Saccon, Joseph M. Dhahbi, Augusto Schneider, Yury O. Nunez Lopez, Ahmad Qasem, Marcelo B. Cavalcante, Lauren K. Sing, Saleh A. Naser, Michal M. Masternak

**Affiliations:** 1 Centro de Desenvolvimento Tecnológico, Universidade Federal de Pelotas, Pelotas 96010-610, Brazil; tatisaccon@gmail.com; 2 Burnett School of Biomedical Sciences, College of Medicine, University of Central Florida, Orlando, FL 32816, USA; ahmadqasem@knights.ucf.edu (A.Q.); marcelocavalcante.med@gmail.com (M.B.C.); saleh.naser@ucf.edu (S.A.N.); 3 Department of Medical Education, School of Medicine, California University of Science & Medicine, San Bernardino, CA 92324, USA; dhahbij@cusm.org (J.M.D.); kimsingl@cusm.org (L.K.S.); 4 Faculdade de Nutrição, Universidade Federal de Pelotas, Pelotas 96010-610, Brazil; augustoschneider@gmail.com; 5 Translational Research Institute, AdventHealth, Orlando, FL 32804, USA; yury.nunez-lopez@adventhealth.com; 6 Department of Obstetrics and Gynecology, Fortaleza University, Fortaleza 60811-905, Brazil; 7 Department of Head and Neck Surgery, Poznan University of Medical Sciences, 61-701 Poznan, Poland

**Keywords:** Crohn’s disease, rheumatoid arthritis, microRNAs, autoimmune diseases, inflammation

## Abstract

**Simple Summary:**

MicroRNAs are small, noncoding RNA, around 23 nt long molecules, expressed in different cell types and involved in post-transcriptional regulation. Importantly, these unique molecules can act directly in cells expressing them, but can also be secreted into blood affecting gene expression in distant or adjacent target cells, thus representing valuable circulating biomarkers of different diseases. In addition, miRNA gene target prediction indicates the important role in pathogenesis of a variety of autoimmune diseases, including Crohn’s disease (CD) and rheumatoid arthritis (RA). Our study of blood circulating miRNAs indicated that there are 99 differentially expressed miRNAs in CD patients and 57 in RA patients, when comparing with healthy individuals. This high number of differentially expressed miRNAs in both autoimmune diseases allowed us to select over 400 different biological processes that were similarly regulated between both groups of patients, which can pave the way to future mechanistic studies for better diagnosis or treatment for patients with Crohn’s disease and rheumatoid arthritis.

**Abstract:**

Crohn’s disease (CD) and rheumatoid arthritis (RA) are immune mediated inflammatory diseases. Several studies indicate a role for microRNAs (miRNAs) in the pathogenesis of a variety of autoimmune diseases, including CD and RA. Our study’s goal was to investigate circulating miRNAs in CD and RA patients to identify potential new biomarkers for early detection and personalized therapeutic approaches for autoimmune diseases. For this study, subjects with CD (*n* = 7), RA (*n* = 8) and healthy controls (*n* = 7) were recruited, and plasma was collected for miRNA sequencing. Comparison of the expression patterns of miRNAs between CD and healthy patients identified 99 differentially expressed miRNAs. Out of these miRNAs, 4 were down regulated, while 95 were up regulated. Comparison of miRNAs between RA and healthy patients identified 57 differentially expressed miRNAs. Out of those, 12 were down regulated, while 45 were up regulated. For all the miRNAs down regulated in CD and RA patients, 420 GO terms for biological processes were similarly regulated between both groups. Therefore, the identification of new plasma miRNAs allows the emergence of new biomarkers that can assist in the diagnosis and treatment of CD and RA.

## 1. Introduction

Immune mediated diseases are a heterogeneous group [1] and defined as a loss of immunological tolerance to self-antigens, resulting in the production of auto-antibodies and subsequent inflammation and tissue damage. These diseases may affect one specific organ or multiple organ systems [2]. Crohn’s disease (CD) is an inflammatory disease affecting any part of the gastrointestinal tract [3]. Symptoms often include persistent diarrhea, abdominal pain, and weight loss [4]. Similarly, rheumatoid arthritis (RA) is a chronic autoimmune inflammatory disease, characterized by chronic joint inflammation, leading to the destruction of bone and cartilage, and reduced functional capacity [5]. Several studies have linked the presence of specific small noncoding RNAs (sncRNA) to CD and RA [6,7,8,9,10,11] and their role in the pathogenesis of a variety of other autoimmune diseases [12].

MicroRNAs (miRNAs) are potent regulators of gene expression and currently the most studied sncRNAs [13]. Furthermore, miRNAs are key players in various biological processes that regulate the differentiation, development, and activation of cells from both innate and adaptive immunity [14,15,16,17]. Circulating miRNAs act in a hormone like fashion, representing an important function for intercellular communication, regulating protein translation in distant or adjacent target cells [18]. Beside their biological role, miRNAs are also valuable molecules as novel biomarkers, predicting a variety of pathological conditions [19]. The dysregulation of miRNAs expression can disturb the immune response, contributing to the pathogenesis of several autoimmune diseases, including CD, RA, lupus and, multiple sclerosis [17]. Several studies demonstrated miRNAs as potential biomarkers for diagnosis and therapeutic of CD and RA; therefore, the discovery and validation of these miRNAs is essential for the improvement of clinical monitoring [6,20,21]. The use of biomarkers can translate basic research into clinical application and be used as a substitute agent for assessing clinical benefits. Therefore, our study’s goal was to investigate circulating miRNAs in CD and RA patients to determine potential new biomarkers for early detection and personalized therapeutic approaches for autoimmune diseases.

## 2. Methods

### 2.1. Patients, Ethical Approval and Plasma Collection

Peripheral blood samples were collected in a 4.0 mL K_2_-EDTA tube from a total of 22 participants, including 7 CD patient samples diagnosed with moderate to severe stage of the disease from the Digestive and Liver Center of Florida, in addition to 8 RA patient samples and 7 healthy control samples acquired from the University of Central Florida Health Center. The average age of CD patients was 42.7 ± 12.4 (22 to 67 years old, 20% females), and the average age for RA patients and healthy controls was 51 ± 13.3 (28 to 73 years old, 90% females) and 25 ± 5.1 (21 to 38 years old, 50% females), respectively. There were no reports of other comorbidities associated with the selected groups of patients. The study was approved by the University of Central Florida Institutional Review Board #IRB00001138. Written consent was completed and signed by each individual before samples were collected.

### 2.2. RNA Isolation and miRNA Library Construction

Blood samples were centrifuged at 3000 RPM for 10 min at room temperature, then 1.0 mL of plasma was transferred to sterile 1.5 mL microtube and stored at −80 °C. The plasma was removed from the freezer and homogenized with QIAzol (Qiagen, Valencia, CA, USA). Total RNA from 200 ul of plasma (*n* = 7 for healthy individuals; *n* = 7 for CD patients; *n* = 8 for RA patients) was extracted using a commercial column purification system (miRNEasy Serum/Plasma kit, Qiagen) following manufacturer’s instructions.

Libraries for miRNAs were prepared using NEXTFlex Small RNA Library Prep Kit v3 (Cat #5132–06), following the manufacturer’s instructions. Briefly, small RNAs were ligated with 3′ and 5′ adapters and reverse transcribed to produce single-stranded cDNAs. For amplification, 14 PCR cycles were used (94 °C for 30 s, 14 cycles of 95 °C for 20 s, 60 °C for 30 s, and 72 °C for 15 s, and a final step of 72 °C for 2 min) with indexes to allow individual processing of each library in single flow cell lane. Following PCR, 6% acrylamide gel was used for sample selection and purification. Finally, the libraries were quantified using BioAnalyzer and RNA Nano Lab Chip Kit (Agilent Technologies, Santa Clara, CA, USA) before being combined into a single microtube for sequencing on a HiSeq 2500 instrument (Illumina Inc., San Diego, CA, USA) using the Illumina HiSeq v4 kit in a single read 50 bp run.

### 2.3. miRNA Sequencing and Statistical Analyses

Sequencing reads were processed with miRDeep2 [20], for removal of adapter sequence and filtering of reads with minimum length of 18 nucleotides. Only reads mapping perfectly to the human genome five times or less were accepted. For miRNA identification, we used the known miRNA input from miRBase v.22.1 [21], and designated *Homo sapiens* as the related species. The miRDeep2 algorithm uses an miRNA biogenesis model to detect known miRNAs and discover novels miRNAs. The miRNA expression values were analyzed with the Bioconductor package edgeR [22] to detect statistically significant changes in circulating miRNAs among groups. The algorithm of edgeR fits a negative binomial model to the count data, estimates dispersion, and measures differences using the generalized linear model likelihood ratio test. The fitted count data were analyzed by performing pairwise comparisons between RA/CD compared to healthy controls to measure the differential. RA was also compared to CD to determine the difference in circulating miRNAs between the two conditions. In addition, *p* values were adjusted for multiple testing using the Benjamini and Hochberg method to control the false discovery rate (FDR).

### 2.4. miRNA Gene Target Prediction, Pathways and Gene Ontology

Target genes of the miRNAs regulated were predicted using the mirPath tool (version 3.0), based on the microT-CDS v. 5.0 database [23]. KEGG pathways and gene ontology (GO) terms for biological processes [24,25] were retrieved using the same tool. Pathways and GO terms with *p* values lower than 0.05 were considered as regulated.

## 3. Results

**Figure 1:**
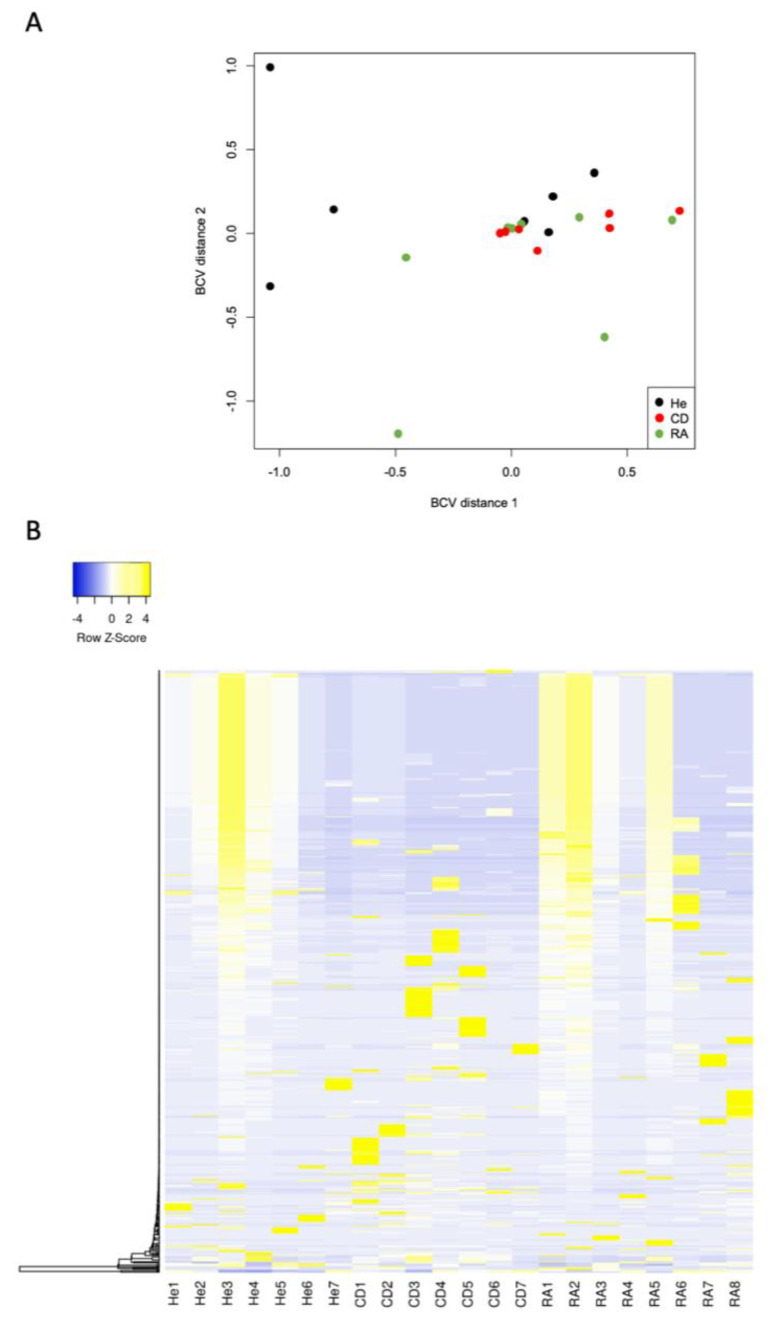
Principal components analysis of the most variable serum miRNAs in Crohn’s disease patients (*n* = 7-red), rheumatoid arthritis patients (*n* = 8-green) and control health patients (*n* = 7-black) (**A**). Unsupervised hierarchical clustering of expression levels of the top miRNAs in serum of Crohn’s disease patients (CD, *n* = 7), rheumatoid arthritis patients (RA, *n* = 8) and control healthy patients (He, *n* = 7) (**B**).

Using PCA analysis, we observed that samples grouped in similar patterns and showed lower variation in axis distances, indicating a low level of sample variability and a significant overlap (Figure 1A). Using unsupervised hierarchical clustering, we could observe that the expression patterns from each group were similar, with some clear signatures among CD and RA (Figure 1B).

**Figure 2:**
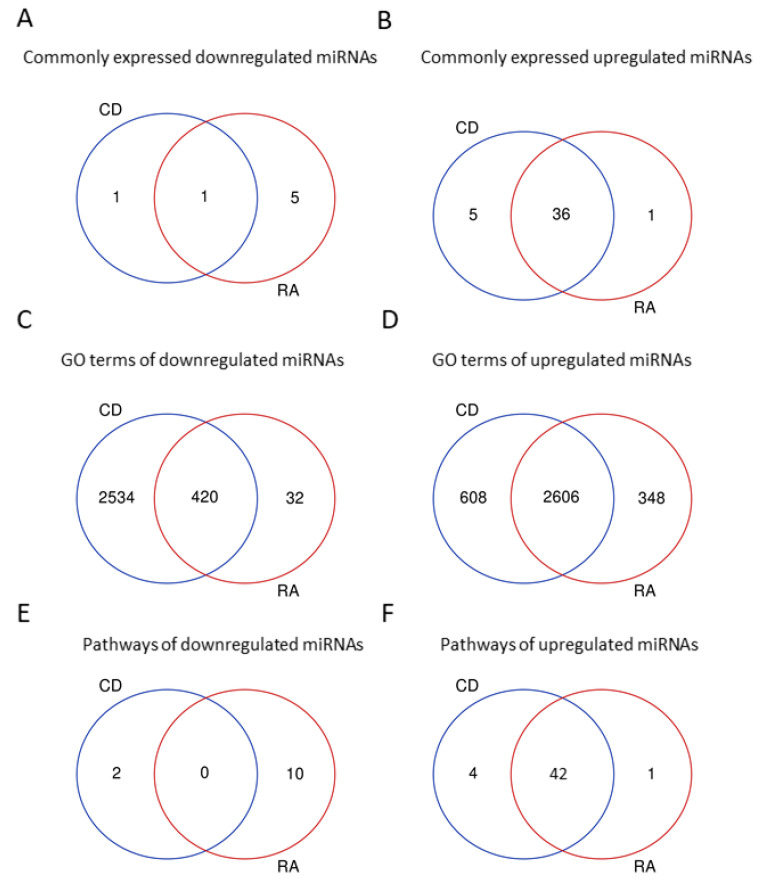
Venn diagram indicating the down regulated (**A**) and up regulated (**B**) miRNAs commonly regulated for RA and CD patients. GO terms for biological processes of down regulated (**C**) and up regulated (**D**) miRNAs and KEGG pathways of down regulated (**E**) and up regulated (**F**) miRNAs in CD and RA patients compared to healthy controls.

We identified 99 differentially expressed miRNAs between CD and healthy patients. Out of these, 4 were down regulated (FDR < 0.05 and FC < 0.5) and 95 were up regulated (FDR < 0.05 and FC >2.0; full list provided in Table S1). Additionally, 24 novel miRNAs were significantly differentially regulated between CD and healthy patients (Table S2), 7 were down (FDR < 0.05 and FC < 0.5) and 17 were up regulated (FDR < 0.05 and FC > 2.0). Between RA and healthy patients, 57 miRNAs were differentially expressed. Out of those, 12 were down (FDR < 0.05 and FC < 0.5), while 45 were up regulated (FDR < 0.05 and FC > 2.0; full list provided in Table S3). Twenty-three novel miRNAs were differentially regulated between RA and healthy patients (Table S4), 4 were down, while 19 were up regulated. Forty-three known miRNAs were commonly regulated in CD and RA patients, 36 up regulated in both CD and RA, 5 up regulated in CD and down regulated in RA and 1 (miR-186-5p) was down regulated in CD and up regulated in RA patients (Table 1; Figure 2A,B). Only mir-2019a-5p was commonly down regulated in both CD and RA patients.

Pathway and GO term enrichment analysis indicated that several important processes are regulated in CD and RA patients. For miRNAs down regulated in CD and RA patients, 42 pathways were commonly up regulated between both groups (Figure 2F), but no pathways were commonly down regulated. The pathways regulated are shown in Table 2 and Table 3. Among commonly regulated pathways, we observed pathways related to the immune system (B-cell activation, T-cell activation, FAS, and Janus kinase/signal transducer and activator of transcription (JAK/STAT) activation pathways), inflammatory response (inflammation mediated by chemokine and cytokine, interferon-gamma, and interleukin signaling pathways), and others related to apoptosis, growth factors, and tumorigeneses.

Regarding GO terms for biological processes, we observed 420 common down regulated terms and 2606 common up regulated terms. The complete list of regulated GO terms is provided in Tables S5 and S6 for up and down regulated for RA patients, respectively, and in Tables S7 and S8 for up and down regulated for CD patients, respectively. Among the common up regulated GO terms, we could observe cytokine production (GO:0001816), positive regulation of cytokine biosynthetic process (GO:0042108), positive regulation of cytokine secretion (GO:0050715), regulation of chemokine biosynthetic process (GO:0045073), regulation of macrophage activation (GO:0043030), macrophage activation (GO:0042116), leukocyte chemotaxis (GO:0030595), and positive regulation of lymphocyte activation (GO:0051251). This further indicates that the regulated miRNAs target processes related to the inflammatory and immune response, as observed for pathways.

## 4. Discussion

In the current study, we were able to identify 99 miRNAs differentially expressed in the plasma of CD patients and 57 miRNAs differentially expressed in the plasma of RA patients. A previous study by Ibora and colleagues found that miR-27a and miR-140-5p were also up regulated in the plasma of CD patients, compared to healthy controls [26]. In another study, 11 miRNAs were found to be regulated in the plasma of CD patients [27]; of those, 4 were also regulated in our study. These two independent studies validated some of our detected miRNAs, indicating common regulated miRNAs in different studied cohorts, regardless of methodological approaches. Beside the partial validation of some previously detected miRNAs by our laboratory, sequencing analysis is a more powerful method for uncovering regulated miRNAs. The overlap between four miRNAs from our study and by Zahm et al. is interesting, as their cohort consisted of pediatric CD patients, while our patients were much older. This indicates that some plasma miRNAs are altered by CD despite patients’ ages. Beside circulating levels of these miRNAs, Wu and colleagues found 13 miRNAs differentially expressed in intestinal biopsies from CD patients [8], of those, six were also found to be regulated in the plasma of CD patients in our study. In addition, 16 miRNAs were differentially expressed in the feces of CD patients [28], six of these miRNAs were also differentially expressed in our study. In contrast, a study with RA patients found 32 miRNAs that were differently expressed in plasma [29] and 2 of these were also regulated in our study. Tseng and collaborators, using synovial fibroblasts of RA patients, found 85 miRNAs differently expressed [30] and 11 of these miRNAs were commonly regulated compared to our study. It is well known that interstitial lung disease (ILD) is frequently associated with RA [31]. A study conducted with RA patients with ILD showed 17 miRNAs differently expressed that were commonly expressed in our study [32]. This evidence shows that miRNAs regulated in the plasma samples of CD and RA from our cohorts are common to previous studies, in different degrees. This further reinforces the existence of a core of miRNAs consistently regulated in autoimmune diseases. Additionally, the overlap of some miRNAs regulated in plasma with miRNAs detected in cells, tissues or even fecal samples from CD and/or RA patients, indicates the potential of easily accessing blood circulating miRNAs as novel biomarkers of these diseases. More importantly, these findings will present future directions, allowing researchers to focus studies on specific genetic pathways targeted by these common miRNAs.

In our study, the top five miRNAs up regulated in CD patients (miR-4433b-5p, miR-126-5p, miR-221-5p, let-7d-5p and miR-3121-5p) are all involved in the regulation of inflammation, and, therefore, may have a functional role in the pathogenesis of the disease. Wang and collaborators [33] showed that miR-4433b-5p is associated with an increased CD44 cell activation, while Feng et al. [34] found that miR-126 was increased in active ulcerative colitis (UC) tissues compared to healthy controls. Additionally, the up regulation of miR-126-5p [34] and miR-221-5p [35] may contribute to the pathogenesis of CD by targeting the nuclear transcription factor kappa B (NFkB), signaling up regulation. Another study examining the effects of antitumor necrosis factor-α (TNF-α) treatment on miRNA expression in the plasma of CD patients found elevated expression of let-7d after 6 weeks of treatment [36]. Although the role of let-7 miRNAs in cellular biology is quite extensive [37], there is strong evidence that this miRNA plays a significant role in the resistance to apoptosis and chronicity of inflammation in CD patients [38,39]. Finally, a study found the strong expression of miR-3121-5p in gut mucosa cells of patients with colorectal cancer, which are characterized by increased signaling of toll like receptor 3 and 8, exacerbating inflammation and inducing tumorigenesis [40]. Therefore, this makes clear the connection between the top regulated miRNA in CD and the inflammatory process, evidencing the nature of this disease. This also suggests the importance of further studies of these miRNAs in different cohorts and, more importantly, that these specific miRNAs may be mechanistically involved in CD pathogenesis.

Similarly, the top five miRNAs up regulated in RA patients (miR-7-5p, miR-9-5p, miR186-5p, miR-2110, and miR-543) have been reported in previous studies with separate cohorts affected by RA. Importantly, both miR-7-5p and miR-543 were commonly observed as up regulated in the synovial fibroblasts of RA patients [30], RA patients with ILD [32] and in our study. Predicted target analysis of miR-7-5p indicate that this miRNA can directly target the TNF-α/NFkB signaling pathway, suggesting a significant impact on inflammatory responses [30,41] and possible mechanistic involvement in the pathogenesis of the disease. Surprisingly, our mir-9-5p data do not corroborate with a previous study in RA patients, in which miR-9-5p was down regulated and correlated with immune/inflammatory cytokines [29]. However, the up regulation of miR-186-5p in our cohort affected by RA agrees with previous findings from independent RA cohorts also characterized by the up regulation of this miRNA [42,43]. A study conducted by Cheng and collaborators [43] showed that miR-186–5p was a predictor of subclinical atherosclerosis. As RA patients have an increased risk of premature mortality compared to the general population, primarily due to an increased incidence of cardiovascular disease [44], miR-186-5p may serve as a biomarker for risk stratification in RA patients with mild to moderate cardiovascular risk. Surprisingly, the up regulation of miR-2110 was not observed in other studies with RA patients or other immune/inflammation conditions. However, miR-2110 up regulation has been related to the proliferation, migration, and invasion of cancer cells [45,46,47,48,49,50]. This further evidences the heterogeneity of this condition and the need for more studies to find consistent regulated miRNAs among individuals. In addition, both CD and RA show the different courses and severities of the disease and these factors might influence how the miRNAs are regulated.

Despite some miRNAs being exclusively regulated in CD or RA patients, forty-two miRNAs were commonly regulated in in these two cohorts. This evidence shows that CD and RA patients share several common circulating miRNAs. Recently, with the development of new techniques such as microarrays and next generation sequencing, large amounts of miRNAs have been identified with a potential role in the pathogenesis of autoimmune diseases [12]. Crohn’s disease is a chronic relapsing inflammatory disorder of the intestine with immunological disturbance [51]. Autoimmune RA is a multisystem disease characterized by immunological abnormalities, affecting mainly the joints and/or connective tissue [52]. An increasing body of evidence demonstrates that CD has a shared genetic background with RA, despite their different target organs [53,54,55]. In recent years, an increasing number of studies have noted that RA tended to cluster with IBD [56,57,58,59] and that patients with IBD are more likely to develop RA [60,61]. Importantly, there is strong evidence for overlap between CD and other autoimmune arthritis conditions, including psoriatic and RA. The genetic studies of different autoimmune diseases showed that mutations in 22 genes are shared by at least two conditions, including CD, ulcerative colitis, psoriasis and RA [62]. Our study points to the same direction, showing 42 miRNAs commonly regulated between these conditions.

Surprisingly, only one miRNA commonly shared between CD and RA was down regulated, hsa-miR-219a-5p. Furthermore, mir-219a has been associated with the development of autoimmune diseases and carcinoma. Importantly, miR-219a was not detected on cerebrospinal fluid from patients diagnosed with multiple sclerosis, compared with healthy individuals with a higher expression of this miRNA [63]. Injection of miR-219a-5p reduces chronic inflammatory pain spinal neuronal sensitization, suggesting the potential use of miR-219a-5p as a future therapeutic agent for treatment of chronic inflammatory pain [64,65]. Beside its significant anti-inflammatory function, miR-219a-5p can inhibit cancer cell proliferation, metastatic activity and epithelial mesenchymal transition by targeting different oncogenes [66,67,68]. These findings suggest the significant role of miR-219a in the suppression of inflammation and cancer development and progression, as well as protection against intestinal mucosal inflammation [69].

Importantly, beside the focus on miRNA differential regulation, the pathways and GO terms enrichment analysis indicated that several processes were similarly regulated between both CD and RA patients. We could observe several pathways and GO terms related to inflammation and immune response up regulated in both CD and RA patients. For example, cellular processes regulated by up regulated miRNAs in the plasma of both CD and RA patients were involved in immune response and inflammation, such as B-cell and T-cell activation, FAS and JAK/STAT activation pathways, inflammation mediated by chemokine and cytokine, interferon-gamma, and interleukin signaling pathways. As both CD and RA are chronic inflammatory autoimmune diseases, it makes sense that miRNAs involved in the up regulation of these pathways were among the ones detected. This suggests that these miRNAs can be involved in the pathogenesis of these diseases by regulating these pathways.

Overall, the signature of regulated miRNAs observed in this study can detect individuals at high risk for RA (miR-499, miR-22 and miR-103a), while others appear to be more directly associated with RA evolution (miR-16, miR-24, miR-125a and miR-223), activity (miR-223), as well as the prediction of response to treatment (miR-125b and miR-223). However, most importantly, several miRNAs have the potential to be new biomarkers for early detection, and future more personalized therapeutic approaches for autoimmune diseases. Therefore, the identification of the new plasma miRNAs described in our study allows the emergence of new candidates for biomarkers that can assist in the diagnosis of treatment of CD and RA. However, it has to be noted that this study has some limitations, mainly with the number of recruited patients, therefore, in the future, more extensive studies are necessary, including not only larger and more specific cohorts of patients, but also the comparison of plasma with biopsies and extensive mechanistic studies. However, the findings presented in this study, along with the overlap of detected miRNA with published studies from other cohorts, provide a significant base for the development of new studies by research teams.

## 5. Conclusions

In summary, our study found that blood specific miRNAs represent highly valuable biomarkers differentiating CD and RA patients from healthy individuals. Therefore, these circulating miRNAs can be potential novel circulating biomarkers allowing noninvasive and frequent testing to track the progression of a variety of autoimmune diseases. Importantly, GO term and pathway enrichment analysis showed that differentially expressed miRNAs target similar biological processes in both groups of patients, representing potential biomarkers for individual risk of disease, evolution, activity and, more importantly, tracking responses to treatment.

## Figures and Tables

**Table 1:** Known miRNAs commonly regulated in plasma of Crohn’s disease (CD) and rheumatoid arthritis (RA) patients compared to healthy controls.

		CD/Healthy		RA/Healthy	
^1^ Mature miRNA	^2^ CPM	^3^ FC	^3^ FDR	^3^ FC	^3^ FDR
Up-regulated					
hsa-let-7b-5p	99,637	21.4	0.0025	9.2	0.0300
hsa-let-7d-5p	4113	440.7	0.000	59.4	0.001
hsa-let-7e-5p	1010	153.7	0.0006	45.8	0.0066
hsa-miR-10b-5p	706	136.0	0.0022	332.5	0.0008
hsa-miR-125b-5p	263	25.4	0.0216	421.2	0.0003
hsa-miR-1260b	1452	333.4	0.0002	147.6	0.0008
hsa-miR-128-1-5p	347	358.6	0.0002	83.1	0.0029
hsa-miR-128-2-5p	347	358.6	0.0002	83.1	0.0029
hsa-miR-130b-5p	373	92.4	0.0039	276.8	0.0010
hsa-miR-143-5p	538	66.8	0.0058	254.0	0.0009
hsa-miR-181a-5p	915	24.7	0.0228	373.8	0.0004
hsa-miR-2110	446	62.4	0.0055	371.8	0.0005
hsa-miR-223-3p	1140	279.0	0.0001	41.8	0.0049
hsa-miR-24-1-5p	616	350.4	0.0003	89.5	0.0029
hsa-miR-24-2-5p	616	350.4	0.0003	89.5	0.0029
hsa-miR-24-3p	691	351.9	0.0002	77.8	0.0027
hsa-miR-26b-5p	274	122.0	0.0010	57.2	0.0046
hsa-miR-30a-5p	245	60.9	0.0039	98.8	0.0020
hsa-miR-320d	123	32.0	0.0022	15.0	0.0163
hsa-miR-328-5p	961	264.9	0.0004	265.5	0.0005
hsa-miR-342-5p	815	122.3	0.0014	268.0	0.0005
hsa-miR-363-5p	291	135.0	0.0019	318.2	0.0007
hsa-miR-375-5p	164	121.9	0.0013	118.0	0.0017
hsa-miR-4433b-5p	2843	509.1	0.0001	102.2	0.0003
hsa-miR-4732-5p	289	150.5	0.0008	62.4	0.0046
hsa-miR-483-5p	1939	410.0	0.0001	263.0	0.0003
hsa-miR-532-5p	78	103.3	0.0004	13.8	0.0353
hsa-miR-574-5p	133	288.5	0.0003	33.0	0.0148
hsa-miR-584-5p	468	208.4	0.0004	53.7	0.0049
hsa-miR-652-5p	1136	292.0	0.0001	181.4	0.0004
hsa-miR-654-5p	145	212.6	0.0004	16.8	0.0436
hsa-miR-874-5p	260	173.9	0.0006	69.1	0.0034
hsa-miR-92b-5p	597	281.9	0.0001	49.2	0.0031
hsa-miR-9-5p	481	42.9	0.0129	539.1	0.0004
hsa-miR-99a-5p	123	31.9	0.0124	180.6	0.0007
hsa-miR-99b-5p	528	150.1	0.0005	166.3	0.0005
hsa-miR-197-5p	298	256.7	0.0001	-	-
hsa-miR-221-5p	1154	457.6	0.0001	-	-
hsa-miR-320e	160	67.7	0.0004	-	-
hsa-miR-382-5p	521	250.4	0.0001	-	-
hsa-miR-432-5p	2428	396.0	0.0001	-	-
hsa-miR-186-5p	249	-	-	468.5	0.00006
Down-regulated					
hsa-miR-219a-5p	243	−970.1	0.0001	−351.2	0.0002
hsa-miR-186-5p	249	−30.1	0.0119	-	-
hsa-miR-320e	160	-	-	−7.2	0.044
hsa-miR-221-5p	1154	-	-	−18.2	0.008
hsa-miR-197-5p	298	-	-	−19.2	0.005
hsa-miR-382-5p	521	-	-	−19.9	0.005
hsa-miR-432-5p	2428	-	-	−25.0	0.003

^1^ Names of mature known miRNAs from miRBase v22 (human GRCh38/hg38 genome) and novel miRNAs predicted by miRDeep2. ^2^ Average known miRNA read counts per million (CPM) computed over all libraries considering the estimated dispersions and the libraries sizes. ^3^ Fold change and FDR for differential expression were computed by EdgeR. Only miRNAs with an interaction |FC| > 2.0 or <0.5 and a FDR < 0.05 are reported.

**Table 2:** Enriched pathways from target genes of up regulated miRNAs from CD and RA compared to health patients.

	CD × Healthy Patients			RA × Healthy Patients		
Commonly KEGG Pathways of Up-Regulated miRNAs	^1^*p* Value	^2^ Genes	^3^ miRNAs	^1^*p* Value	^2^ Genes	^3^ miRNAs
Alzheimer disease–amyloid secretase pathway	<0.0001	67	13	0.0001	67	9
Alzheimer disease–presenilin pathway	<0.0001	124	22	<0.0001	124	22
Androgen/estrogen/progesterone biosynthesis	0.00132	12	4	0.012	12	3
Angiogenesis	<0.0001	172	43	<0.0001	172	34
Angiotensin II-stimulated signaling through G proteins and beta-arrestin	0.00275	39	6	0.043	39	4
Apoptosis signaling pathway	<0.0001	115	27	<0.0001	115	23
B cell activation	<0.0001	70	20	<0.0001	70	15
Cadherin signaling pathway	0.00341	160	13	<0.0001	160	15
CCKR signaling map	<0.0001	172	59	<0.0001	172	40
Cell cycle	<0.0001	22	7	0.0001	22	6
Cytoskeletal regulation by Rho GTPase	0.0024	83	9	0.039	83	6
EGF receptor signaling pathway	<0.0001	136	30	<0.0001	136	19
Endothelin signaling pathway	<0.0001	82	13	<0.0001	82	13
FAS signaling pathway	<0.0001	35	10	0.034	35	4
FGF signaling pathway	<0.0001	121	25	<0.0001	121	16
Gonadotropin-releasing hormone receptor pathway	<0.0001	232	51	<0.0001	232	37
Hedgehog signaling pathway	0.0002	22	6	0.001	22	5
Huntington disease	<0.0001	143	16	0.0018	143	11
Hypoxia response via HIF activation (P00030)	0.00019	32	7	<0.0001	32	8
Inflammation mediated by chemokine and cytokine signaling pathway	<0.0001	255	43	<0.0001	255	24
Insulin/IGF pathway-mitogen activated protein kinase kinase/MAP kinase cascade	<0.0001	31	13	<0.0001	31	10
Insulin/IGF pathway-protein kinase B signaling cascade	<0.0001	39	14	<0.0001	39	12
Integrin signalling pathway	<0.0001	191	28	<0.0001	191	22
Interferon-gamma signaling pathway	<0.0001	31	15	<0.0001	31	7
Interleukin signaling pathway	<0.0001	88	31	<0.0001	88	21
JAK/STAT signaling pathway	<0.0001	17	9	0.032	17	3
Notch signaling pathway	0.00027	45	8	0.0028	45	6
Oxidative stress response	<0.0001	55	11	0.0072	55	6
p38 MAPK pathway	<0.0001	40	10	<0.0001	40	8
p53 pathway	<0.0001	87	24	<0.0001	87	20
p53 pathway by glucose deprivation	<0.0001	22	7	0.0082	22	4
p53 pathway feedback loops 2	<0.0001	50	26	<0.0001	50	20
Parkinson disease	<0.0001	98	11	0.0018	98	9
PDGF signaling pathway	<0.0001	145	37	<0.0001	145	27
PI3 kinase pathway	<0.0001	52	15	<0.0001	52	13
Plasminogen activating cascade	0.00045	18	4	0.035	18	3
Ras Pathway	<0.0001	74	27	<0.0001	74	19
T cell activation	<0.0001	88	22	<0.0001	88	16
TGF-beta signaling pathway	<0.0001	98	21	<0.0001	98	16
Toll receptor signaling pathway	<0.0001	57	13	<0.0001	57	11
VEGF signaling pathway	<0.0001	68	17	<0.0001	68	18
Wnt signaling pathway	<0.0001	317	37	<0.0001	317	33
KEGG pathways of up-regulated miRNAS in CD patients only						
Axon guidance mediated by Slit/Robo	0.0005	27	6	-	-	-
BMP/activin signaling pathway	0.0093	3	2	-	-	-
GBB signaling pathway	0.0571	2	2	-	-	-
MYO signaling pathway	0.057	2	2	-	-	-
KEGG pathways of up-regulated miRNAS in RA patients only						
Dopamine receptor mediated signaling pathway	-	-	-	0.035	57	5

^1^ Only pathways with *p* values lower than 0.05 were considered as significant; ^2^ Number of genes affected in the pathway by the regulated miRNAs; ^3^ Number of miRNAs differentially expressed that have a target gene in the pathway.

**Table 3:** Enriched pathways from target genes of down regulated miRNAs from CD and RA compared to healthy patients.

	CD × Health Patients			RA × Health Patients		
KEGG Pathways of Down-Regulated miRNAs	^1^*p* Value	^2^ Genes	^3^ miRNAs	^1^*p* Value	^2^ Genes	^3^ miRNAs
CCKR signaling map	<0.0001	112	2	-	-	-
Interleukin signaling pathway	0.0006	88	3	-	-	-
Toll receptor signaling pathway	-	-	-	<0.0001	57	5
p53 pathway feedback loops 2	-	-	-	<0.0001	50	4
Hypoxia response via HIF activation	-	-	-	0.0007	32	2
Insulin/IGF pathway-protein kinase B signaling cascade	-	-	-	0.0011	39	2
p38 MAPK pathway	-	-	-	0.0011	40	2
p53 pathway	-	-	-	<0.0001	87	4
B cell activation	-	-	-	0.0033	70	2
EGF receptor signaling pathway	-	-	-	0.0005	136	3
PDGF signaling pathway	-	-	-	0.0007	145	3
Inflammation mediated by chemokine and cytokine signaling pathway	-	-	-	<0.0001	255	5

^1^ Only pathways with *p* values lower than 0.05 were considered as significant. ^2^ Number of genes affected in the pathway by the regulated miRNAs. ^3^ Number of miRNAs differentially expresses that have a target gene in the pathway.

## Data Availability

The data that support the findings of this study are available from the corresponding author upon request.

## Supplementary Materials

The following supporting information can be downloaded at https://www.mdpi.com/article/10.3390/biology11040508/s1, Table S1. miRNAs differentially expressed in plasma of Crohn’s disease (CD) patients compared to healthy controls, Table S2. Novel miRNAs differentially expressed in plasma of Crohn’s Disease (CD) patients compared to healthy controls, Table S3. miRNAs differentially expressed in plasma of Rheumatoid Arthritis (RA) patients compared to healthy controls, Table S4. Novel miRNAs differentially expressed in plasma of Rheumatoid Arthritis (RA) patients compared to healthy controls, Table S5. Gene ontology (GO) terms for miRNAs up-regulated in plasma of Rheumatoid Arthritis (RA) patients compared to healthy controls, Table S6. Gene ontology (GO) terms for miRNAs down-regulated in plasma of Rheumatoid Arthritis (RA) patients compared to healthy controls, Table S7. Gene ontology (GO) terms for miRNAs up-regulated in plasma of Crohn’s disease (CD) patients compared to healthy controls, Table S8. Gene ontology (GO) terms for miRNAs down-regulated in plasma of Crohn’s disease (CD) patients compared to healthy controls.
