# Supplementary material for: Plasma miRNA Profile of Crohn’s Disease and Rheumatoid Arthritis Patients
Source: Biology (Basel). 2022 Mar 25;11(4):508. doi: 10.3390/biology11040508 (PMC9033111; doi:10.3390/biology11040508)
Supplement: Supplementary file 1 [file biology-11-00508-s001.zip › supplementary/biology-1621436-non_published_material.pdf]

Supplementary Material

Table S1. miRNAs differentially expressed in plasma of Crohn's disease (CD) patients compared to healthy controls.

Table S2. Novel miRNAs differentially expressed in plasma of Crohn's Disease (CD) patients compared to healthy controls.

Table S3. miRNAs differentially expressed in plasma of Rheumatoid Arthritis (RA) patients compared to healthy controls.

Table S4. Novel miRNAs differentially expressed in plasma of Rheumatoid Arthritis (RA) patients compared to healthy controls.

Table S5. Gene ontology (GO) terms for miRNAs up-regulated in plasma of Rheumatoid Arthritis (RA) patients compared to healthy controls.

Table S6. Gene ontology (GO) terms for miRNAs down-regulated in plasma of Rheumatoid Arthritis (RA) patients compared to healthy controls.

Table S7. Gene ontology (GO) terms for miRNAs up-regulated in plasma of Crohn's disease (CD) patients compared to healthy controls.

Table S8. Gene ontology (GO) terms for miRNAs down-regulated in plasma of Crohn's disease (CD) patients compared to healthy controls.
